# Semi-Immediate Autotransplantation of a Maxillary Third Molar With Unfavorable Root Anatomy: A Case Report

**DOI:** 10.1155/crid/6655016

**Published:** 2025-07-04

**Authors:** Juraj Marton, Radovan Žižka, Rami Dabdoub, Zdeněk Pokorný

**Affiliations:** Institute of Dentistry and Oral Sciences, Faculty of Medicine and Dentistry, Palacký University Olomouc, Olomouc, Czech Republic

**Keywords:** CARP, case report, challenging anatomy, delayed, tooth autotransplantation

## Abstract

Tooth autotransplantation is a procedure in which a donor tooth is transplanted within the same patient's jaw to replace a missing tooth. Donor tooth root morphology and periodontal ligament integrity are key factors influencing success. We report a semi-immediate autotransplantation of a maxillary third molar with a 90° divergent root into a site previously affected by a periapical abscess. After the extraction of the compromised tooth (#15) and removal of the interradicular septum, the site was left to heal to allow soft tissue closure and infection resolution. During this period, CBCT imaging and a 3D-printed donor tooth replica (CARP model) were used to plan the procedure, including the intended root amputation. Two weeks later, autotransplantation was performed. The recipient site only required soft tissue and granulation tissue management, with no additional bone preparation, allowing for a minimally traumatic approach. The donor tooth was transplanted with an extraoral time of under 6 min. At the 18-month follow-up, the tooth remained functional, asymptomatic, and radiographically stable. This case highlights the feasibility of delayed autotransplantation following infection and the clinical value of combining imaging with prototyping in surgical planning—particularly when dealing with donor teeth with unfavorable root anatomy.

## 1. Introduction

Tooth autotransplantation is a surgical procedure for replacing a nonrestorable or missing tooth using a donor tooth from the same patient's jaw. The highest success rates are typically achieved with single-rooted premolars exhibiting incomplete root development, due to their more favorable anatomy and greater regenerative capacity [1–3]. In contrast, donor teeth with complex root morphology—such as multirooted molars with divergent roots—pose greater risks [4–7]. These include increased chances of periodontal ligament (PDL) damage during extraction, more difficult fitting into the recipient socket, and complications in endodontic management. Despite these challenges, successful autotransplantation involving donor teeth with complex root anatomy has been documented when meticulous planning and technique are applied [8–11].

The timing of the procedure may also play a role in decision-making. Most literature distinguishes between immediate transplantation (performed on the day of recipient tooth extraction) and conventional transplantation (into fully healed socket) [12]. However, cases that fall between these two categories—termed “delayed” by Tsukiboshi et al. [13] and referred here as semi-immediate—remain underreported. There is little data specifically addressing transplantation into previously infected sites after short healing intervals, and the risks or benefits of this timing are not well understood.

In this case report, we present a case that combines several uncommon factors: a maxillary third molar with a 90° divergent root used as the donor tooth, a recipient site affected by a periapical abscess, and the transplantation performed within 2 weeks after extraction, allowing for soft tissue healing. Preoperative CBCT imaging and computer-aided rapid prototyping (CARP) model enabled detailed planning, including the intentional amputation of the divergent root. The procedure was completed with minimal trauma and a short extraoral period. This case could contribute to the limited literature on semi-immediate transplantation following infection and highlights the potential of advanced planning tools in managing anatomically complex donor teeth.

## 2. Materials and Methods

This case is reported according to the 2013 CARE Checklist.

### 2.1. Primary Situation and Treatment Planning

**Figure 1:**
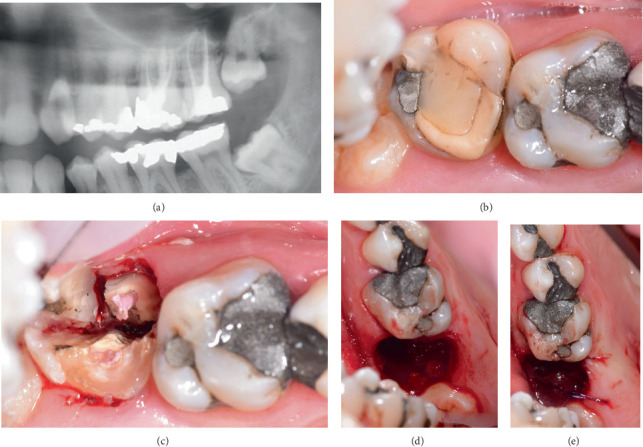
Preoperative situation. (a) The panoramic x-ray shows Tooth #15 with periapical radiolucency and a vertically placed wisdom tooth with suspected fused conical roots. (b) Clinical examination revealed a submucosal abscess with a fistula and Tooth #15 with insufficient postendodontic treatment and secondary caries. (c–e) The tooth was meticulously extracted after sectioning the roots.

**Figure 2:**
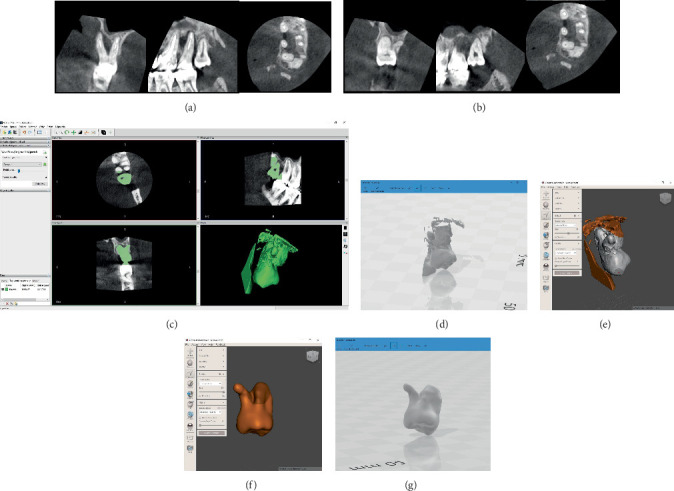
Preoperative planning. The CBCT examination revealed the (a) failed Tooth #15 and (b) donor Tooth #16 with multiple conical fused roots and a buccally placed root measuring 3 mm, angled at 90°. (c–g) The replica was crafted after sectioning the tooth from the CBCT scan and smoothing its surface.

The 31-year-old male patient was being treated at the periodontology department at the faculty hospital in Olomouc due to generalized chronic periodontitis (Stage III Grade B) [14] and multiple gingival recessions. The patient came to the clinic in December 2022 as an acute patient, presenting with pain in the upper left quadrant. Aside from dental issues, the patient had no significant medical history, was in good health, did not take any long-term medication, and had no allergies. The clinical examination of Tooth #15 revealed a periapical abscess with the sinus tract extending to the hard palate (Figure 1b). The underlying cause was attributed to the second molar (Tooth #15), which presented an insufficient postendodontic treatment and secondary caries. Furthermore, the tooth exhibited signs of a periapical lesion and second-degree furcation involvement (Figure 2a), as determined by Hamp et al. [15]. Considering these factors, extraction of the tooth was deemed necessary.

The patient was offered an autotransplantation of the nonoccluding wisdom tooth to replace the extracted second molar. According to the panoramic x-ray (Figure 1a), the donor tooth had fused conical roots, making it potentially suitable for this procedure. Although previously indicated for extraction, the wisdom tooth was retained for potential autotransplantation in the event of second molar failure. The patient consented to the proposed treatment plan, and preoperative CBCT diagnostics were conducted.

The CBCT revealed a vertically positioned wisdom tooth in infraocclusion with complex anatomy (Figure 2b). The mesiodistal and vertical dimension of the crown and the root length and width was measured against the recipient. However, the tooth displayed multiple conical fused roots and a buccally placed root measuring 3 mm in length, angled at 90°. Standard assessment deemed the tooth unsuitable as a donor. Given the surgeon's prior successes with autotransplantation involving teeth with challenging root anatomy, the case was discussed with an endodontist to explore the possibility of RCT. Four root canals were observed on axial sections (Figure 2b), and the endodontist deemed RCT feasible. Subsequently, a semi-immediate surgical procedure was scheduled within the next 14 days to extract the tooth with a planned fracture of the divergent root and retrograde filling of the root canal. The patient consented to participate in the trial and signed an informed consent form, agreeing to the proposed procedure as well as future publication.

The compromised Tooth #15 was carefully extracted after the roots were separated (Figures 1c, 1d, and 1e). Pus was drained through the extraction socket, and to facilitate the semi-immediate autotransplantation, the surgeon carefully removed the interradicular septum between the buccal roots using rongeur forceps. The wound was then left to undergo spontaneous healing of the soft tissues over the course of 2 weeks.

During this interim period, a CARP model of the donor tooth was meticulously crafted, as illustrated in Figures 2c, 2d, 2e, 2f, and 2g. Subsequently, a replica was generated using a 3D printer (Objet30 Dental Prime, Stratasys, 7665 Commerce Way Eden Prairie, Minnesota, United States).

### 2.2. Surgical Procedure

**Figure 3:**
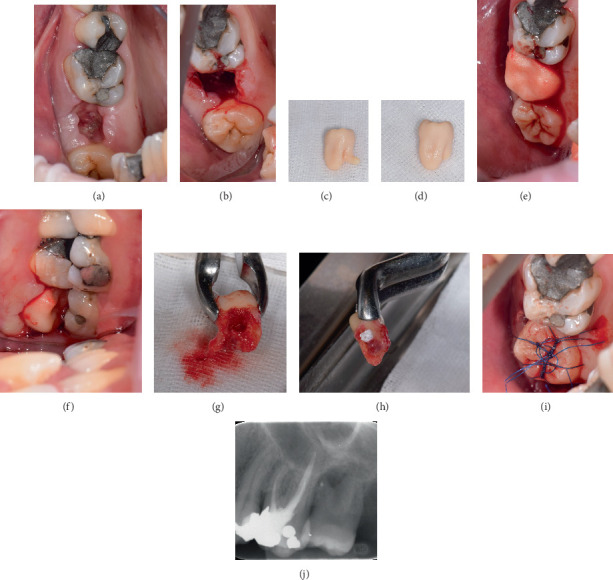
Surgical procedure. (a) The wound healing 2 weeks after the extraction prior to surgery. (b) The preparation of the recipient socket just by excision of granulation tissue. (c, d) The CARP model was adjusted according to the assumed fracture line, and (e, f) the position inside the socket was checked. (g) The donor tooth was atraumatically extracted, utilizing forceps only, and the buccal root remained intact and was removed using a diamond bur in a slow handpiece. (h) The root canal was shaped using an ultrasonic diamond-coated instrument and filled with MTA. A suspected area 0.8 × 1 mm devoid of PDL at the opposite site was visible. (i) The tooth was placed in the recipient socket and secured by suture. (j) Immediate postoperative periapical radiograph.

The surgical procedure was conducted at the end of December 2022. On the day of the surgery, the surrounding soft tissues at the place of the extraction socket of Tooth #15 were healed, with no evidence of prior inflammation (Figure 3a). Retromaxillary tuber anesthesia was administered using 4% articaine (Supracain, Zentiva, Prague, Czech Republic). The recipient socket was prepared solely by a circular incision at the top of the alveoli, with an excision of the granulation tissue within the previous socket (Figure 3b). Thanks to the previous interradicular septum removal, no additional osteotomy was needed. This approach facilitated the optimal cervical adaptation of the soft tissues, avoiding additional preparation trauma to the recipient socket. The CARP model was adjusted to facilitate the cutting of the divergent root using a diamond drill along the assumed fracture line (Figure 3c,d). Subsequently, the fit of the replica was assessed within the recipient socket, achieving a position in infraocclusion without the need for drilling adjustment (Figure 3e,f).

The donor tooth was atraumatically extracted after a sulcular incision, utilizing forceps exclusively in a pulling motion along the long axis of the tooth. The buccal root remained intact during the procedure, which was not anticipated. The surgeon promptly evaluated the root surface, revealing a suspected 0.8 × 1 mm area devoid of PDL at the opposite site (Figure 3g). This area was viable for healing without complications, with the potential for PDL regeneration. Following this assessment, the divergent root was removed using a diamond bur (Meisinger, Neuss, Germany) in a slow handpiece. The root canal was shaped using an ultrasonic diamond coated instrument (Woodpecker Medical Instruments Co., Guilin, Guangxi, China), followed by a filling with MTA (Well Root PT, Vericom, Chuncheon-si, Gangwon-do, Korea) (Figure 3h). Throughout the procedure, the tooth was kept in saline-soaked gauze. The total extraoral time of the donor tooth was 5 min and 20 s before it was placed in the recipient socket. The position of infraocclusion was maintained, and the tooth was subsequently secured in the socket using synthetic nonabsorbable sutures, as depicted in Figure 3i (Mopylen 5/0, Resorba Medical GmbH, Nürnberg, Gremany). An immediate postoperative periapical radiograph confirmed the positioning (Figure 3j).

The patient received postoperative instructions to refrain from brushing the area for 1 week and was prescribed 1 g of amoxicillin every 12 h for 7 days (Amoksiklav, Sandoz, Switzerland).

### 2.3. Postoperative Care and RCT

The postoperative follow-up was performed 1 and 4 weeks after the surgery. Seven days after the surgery, the tooth exhibited primary healing of the soft tissues (Figure 3a). The sutures were removed. Due to the tooth's high mobility, the surgeon applied a wire-resin splint according to the Tsukiboshi protocol [13]. By the fourth week, the tooth demonstrated no pathological mobility, pain on percussion, or pathological sounds, and the splint was removed.

**Figure 4:**
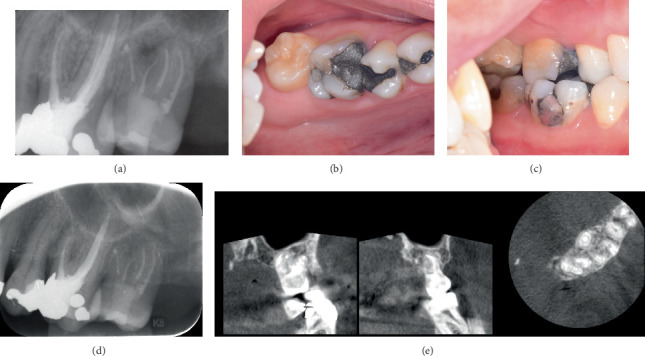
The postoperative care and final follow-up. (a) A periapical radiograph was taken on the day of the root canal treatment. (b, c) The final follow-up, conducted 18 months after the surgery, confirmed that the tooth remained functional and clinically asymptomatic, without the need for additional restoration. (d) Radiographic examination revealed no signs of complications. (e) Additionally, a CBCT scan was conducted to properly evaluate the healing of both the resected root and the previously damaged root surface.

At the beginning of February 2023, the endodontist performed elective RCT of the tooth (Figure 4a). No pathological clinical findings were observed in the tooth (Figure 3b). Local anesthesia of 2 mL of 4% articaine (Supracain, Zentiva, Prague, Czech Republic) was administered, and four root canals were shaped using the ProTaper Gold system (Maillefer, Encublens, Switzerland) up to the physiological apex. The length was verified using an electric apex locator (Woodpecker Ai-Pex, Guilin Woodpecker Medical instruments Co., Guilin, Guangxi, China). The irrigation protocol utilized 5% NaOCl with ultrasonic activation and 23% EDTA. Subsequently, obturation was accomplished using individualized ProTaper Next Conform Fit Gutta-Percha (Maillefer, Encublens, Switzerland) and BioRoot RCS (Septodont, Saint-Maur-des-Fossés, France).

## 3. Results

The patient underwent follow-up examinations every 6 months while being treated for periodontal disease and mucogingival recessions. During routine examinations, a regular radiological examination involving periapical radiographs was performed. The most recent check-up occurred 1.5 years after the autotransplantation in May 2024.

Clinically, the tooth was asymptomatic and exhibited a negative reaction to the percussion test. The tooth's mobility was assessed using the Periotest M device (Medizintechnik Gulden, Modautal, Germany) (Figures 4b, 4c, and 4d). With the patient's permission, a CBCT scan was conducted at the most recent check-up (Figure 4e) to properly evaluate the healing around the resected root and the previously damaged root surface. The autotransplantation was deemed successful.

The patient was satisfied with the outcome of the tooth autotransplantation and with the whole periodontal treatment. Satisfied with previous periodontal treatment, he trusted the treatment planning and gave consent for the trial and publication. At the last check-up, the patient also allowed for a check-up CBCT scan to properly evaluate the healing around the resected root and the previously damaged root surface.

The timeline from the diagnosis to the last check-up is shown in Table 1.

## 4. Discussion

The term semi-immediate tooth autotransplantation, referring to transplantation performed within several days to a few weeks after extraction, is not commonly used in the scientific literature. Surgical timing is traditionally categorized as either immediate transplantation, performed on the same day as the extraction at the recipient site, or conventional transplantation, involving placement into a surgically crafted socket after complete healing [12]. The interval between these two approaches—termed delayed by Tsukiboshi et al. [13]—aims to allow for soft tissue healing while reducing the need for invasive socket preparation. However, references to this approach remain limited, and both the terminology and timeframes are inconsistently defined across publications [16, 17].

In the presented case, the semi-immediate approach was selected due to an acute condition requiring extraction, which manifested as a submucosal abscess with a palatal fistula. The 14-day healing period facilitated soft tissue closure and the resolution of inflammation while also providing sufficient time for detailed preoperative planning, including the fabrication of a CARP model. The interradicular septum was removed at the time of the extraction of the compromised tooth, which subsequently allowed for a simplified preparation of the recipient site limited to granulation tissue removal, without the need for any additional ostectomy. This contributed to precise donor fitting (first tested using the CARP model), a reduced surgical time, and overall minimal procedural trauma.

Donor teeth with complex root anatomy—such as multirooted molars with divergent roots—carry a higher risk due to the increased likelihood of root surface damage during extraction, more difficult adaptation to the recipient site, and prolonged extra-alveolar time. These factors significantly increase the risk of PDL damage, which may lead to root resorption, ankylosis, or transplant failure [4–7]. It has been proposed that areas devoid of PDL exceeding 1 × 1 mm^2^ may compromise regeneration on the damaged surface [18]. In this case, the surgeon visually assessed the damaged root surface and estimated that the area is below the threshold, justifying the expectation of PDL growth. Throughout the follow-up period, periapical radiographs showed no signs of root resorption, and Periotest measurements consistently reflected normal functional mobility. This clinical assessment was subsequently confirmed by CBCT imaging at the final follow-up.

In addition, complex root morphology often involves challenging root canal anatomy, complicating RCT. The complicated shapes and multiple canals of multirooted teeth reduce the likelihood of endodontic success, particularly after transplantation. CBCT imaging improves feasibility of RCT in such cases by providing accurate visualization of canal number and configuration [19]. For these reasons, the most favorable outcomes in tooth autotransplantation are typically reported in premolars with incomplete root formation [1, 2]. Despite these challenges, multiple case reports have documented successful autotransplantation of donor teeth with complex root anatomy when meticulous planning and refined technique are applied [8–11]. In the presented case, the primary challenge was the donor tooth's 90° divergent root. CBCT imaging enabled detailed assessment of the donor tooth's morphology, allowing for preoperative planning that included intentional root amputation. The use of the CARP model further enhanced surgical precision and enabled precise recipient site preparation.

This case illustrates how the semi-immediate approach can be beneficial in situations where immediate autotransplantation may carry risks due to unresolved inflammation or complex donor anatomy. In this report, the semi-immediate approach is defined as transplantation performed within 14 days after extraction, allowing sufficient soft tissue healing while avoiding the need for extensive socket preparation. This interval also provides time for meticulous preoperative planning, including CBCT assessment and CARP model fabrication, which contributed to the successful outcome despite challenging root morphology. The primary limitation remains the single-case nature of the report; broader evaluation through clinical studies is necessary to assess the generalizability of this approach. Furthermore, in cases where an immediate approach is feasible, the additional delay may not offer any significant advantage.

## 5. Conclusions

As demonstrated in the presented case, semi-immediate autotransplantation may be a viable option when immediate transplantation is contraindicated due to infection or complex donor anatomy. The favorable outcome underscores the value of advanced planning tools, such as CBCT imaging and CARP models, in enabling successful management of challenging cases. However, this report presents a single case, and further clinical studies are necessary to establish the optimal timing and indications for the delayed approach in broader clinical practice.

## Figures and Tables

**Table 1:** Timeline.

**Date**	**Procedure**
22.11.2022	Diagnosis, extraction of Tooth #15
28.11.2022	3D model crafting
5.12.2022	CARP model printing
6.12.2022	Surgery—Tooth autotransplantation
13.12.2022	Postoperative check-up, wire-resin splint
3.1.2023	Check-up, splint removal
7.2.2023	Root canal treatment (RCT)
3.3.2023	Check-up after RCT
28.7.2023	Recall
14.3.2024	(Mucogingival surgery, lower incisors)
3.4.2024	(Postoperative check-up)
31.5.2024	Recall (most recent check-up)

## Data Availability

The data that support the findings of this study are available from the corresponding author upon reasonable request.
